# Quantum-Size Effects in Ultra-Thin Gold Films on Pt(111) Surface

**DOI:** 10.3390/ma17010063

**Published:** 2023-12-22

**Authors:** Yury M. Koroteev, Igor V. Silkin, Vyacheslav M. Silkin, Evgueni V. Chulkov

**Affiliations:** 1 Institute of Strength Physics and Materials Science, Siberian Branch of Russian Academy of Sciences, 634055 Tomsk, Russia; 2 Laboratory of Electronic and Spin Structure of Nanosystems, Saint Petersburg State University, 198504 Saint Petersburg, Russia; 3 Faculty of Physics, Tomsk State University, 634050 Tomsk, Russia; 4 Departamento de Polímeros y Materiales Avanzados: Física, Química y Tecnología, Facultad de Ciencias Químicas, Universidad del País Vasco (UPV-EHU), Apdo. 1072, 20080 San Sebastián, Spain; 5 Donostia International Physics Center (DIPC), P. Manuel Lardizabal 4, 20018 San Sebastián, Spain; 6 IKERBASQUE, Basque Foundation for Science, Pl. Euskadi 5, 48009 Bilbao, Spain; 7 Centro de Física de Materiales (CFM-MPC), Centro Mixto CSIC-UPV/EHU, P. Manuel Lardizabal 5, 20018 San Sebastián, Spain

**Keywords:** adsorbates, thin metal films, quantum well states, surface states

## Abstract

We calculate, within the density-functional theory, the atomic and electronic structure of the clean Pt(111) and Au(111) surfaces and the *n*ML-Au/Pt(111) systems with *n* varying from one to three. The effect of the spin–orbital interaction was taken into account. Several new electronic states with strong localization in the surface region were found and discussed in the case of clean surfaces. The Au adlayers introduce numerous quantum well states in the energy regions corresponding to the projected bulk band continuum of Au(111). Moreover, the presence of states resembling the true Au(111) surface states can be detected at *n* = 2 and 3. The Au/Pd interface states are found as well. In *n*ML-Au/Pt(111), the calculated work function presents a small variation with a variation of the number of the Au atomic layer. Nevertheless, the effect is significantly smaller in comparison to the *s*-*p* metals.

## 1. Introduction

The properties of atomically thin metallic films can be very different from those of the corresponding bulk crystals. This change is generally considered to be a quantum-size effect (QSE). For the first time, the phenomena, related to finite film thicknesses, were observed experimentally in Refs. [1,2]. Due to QSE, the continuous bulk energy bands split up into discrete energy states in the direction perpendicular to the film plane [3]. These discrete thin-film states, often termed quantum well states (QWSs), are generated in the wave-vector–energy range determined by respective bulk energy bands. The number of such states depends on the number of atomic layers in the film. Thin-film QWSs can be directly observed by photoemission, inverse photoemission, and two-photon photoemission [3,4,5,6,7,8,9,10,11,12], as well as by scanning tunneling microscopy/spectroscopy (STM/STS) [13,14,15] experiments.

QWSs play an important role in various areas of condensed matter physics. Such states are at the origin of many features, including those that demonstrate oscillating behavior with the film thickness. Among them are the electron-phonon coupling [16,17,18], interlayer distances [19], preferred island heights [20], binding energies [21,22], quasiparticle lifetimes [23,24,25], work functions [26,27,28,29,30], energetic stability of metal thin films [3,28,31,32], surface energies [33], plasmonic modes in thin metallic films [34,35,36,37,38], chemical activity [39,40], etc. The chemical properties of overlayers can also be strongly modified in connection with the atomic thickness of the overlayer as compared to the bulk material surfaces.

A particle-in-box simple picture based on a jellium model works reasonably well for QWSs in the case of simple metals. Usually, in this case, the amplitude of all QWSs is similar in the vicinity of all atomic layers composing the film. For *s*-*p* metals, the resulting QWSs have almost free-electron-like dispersion in the plane parallel to the surface. This picture was observed, i.e., in the case of Pb [24,41]. Essentially, the same situation is realized in the case of electronic states around the Fermi level in noble metals like Cu, Au, and Au since the *d* band is totally occupied [3]. In this case, QWSs of a film were used to sample the electronic structure of the parent bulk materials [42].

This picture can be complicated by several factors. Thus, electrons in the adsorbed thin metal films are confined to the asymmetrical potential well formed by the vacuum barrier on one side and the absolute or symmetry energy band gap of the substrate on the opposite side. The influence of the substrate can be rather important. Especially strong effects are expected in ultra-thin films due to the relative strength of the adlayer/substrate interface potential. Moreover, depending on the mismatch between the energy gaps of the adlayer and the substrate, the QWSs can be of two types: true electronic states, fully confined in the film due to the substrate energy gap, and the resonances that couple to the substrate bulk bands. Formation of surface states [43,44,45] in the adlayer and evolution of those of the substrate upon adlayer deposition is another issue to address. In the case of transition metals with flat *d*-type energy bands, the situation may change as well. Instead of a uniform distribution of QWSs over the film, the resulting QWSs of *d* type may present strong localization up to about 100% in some atomic layers [46].

Among many heterostructures studied up to now, the Au/Pt(111) system attracts special interest. Platinum is widely employed as a catalytic material in the chemical industry. In particular, various properties of atomically clean platinum surfaces were studied in detail both experimentally and theoretically. On the other hand, studies of bimetallic surfaces involving gold adlayers have revealed a very interesting chemistry of such systems. Gold has long been regarded as an “inert” material in many chemical reactions. However, these well-known properties of bulk gold tend to change at nanometer size. For instance, when the Au particle size decreases down to 5–10 nm, the gold shows high activity as a catalyst [47]. A rather comprehensive review of the gold catalytic properties can be found in [48]. Gold films on the Pt(111) surface grow layer-by-layer for several atomic layers [49,50,51,52]. A detailed description of the growth process can be found elsewhere [53].

The properties of the Au/Pt(111) adsorbate system were investigated experimentally and theoretically for several decades [54,55,56,57]. Photoemission studies were performed for different Au coverage [49,50,58]. First-principles calculations of the electronic structure for one Au ML were made as well [52,58]. The details of how the Shockley-like surface state of Pt(111) evolves upon adsorption of ultra-thin gold layers were reported in [59]. However, to the best of our knowledge, a systematic study of the electronic properties of gold adlayers of a few MLs thickness on Pt(111) has not been performed yet.

Here, we present a detailed theoretical investigation to understand the properties of Au films on Pt(111). In particular, we study the atomic structure of the *n*ML-Au/Pt(111) heterostructures. Also, we study the variation in the electronic structure of the Pt(111) surface upon deposition of several atomic layers of gold. Special focus is made on the formation of the gold-induced quantum well and surface states in such atomically thin adsorbate systems. The impact of spin-orbit interaction on the electronic states is studied as well. Finally, the trends in the work function in the *n*ML-Au/Pt(111) systems are analyzed.

## 2. Calculation Method

The results in this work were obtained within the framework of the first-principle approach based on the density-functional theory using all-electron full-potential linearized augmented plane-wave (FP-LAPW) method [60], as implemented in the FLEUR code [61]. This implementation allows us to calculate the total energy and forces acting on the atoms, which is necessary to optimize the lattice parameters and the position of atoms in the unit cell. The generalized gradient approximation (GGA) in the parametrization of Perdew–Burke–Ernzerhof (PBE) [62] was used for the description of the exchange-correlation potential. The core states were treated fully relativistically, while the valence states were computed in a scalar relativistic approximation, both with spin-orbit coupling (SOC) and without SOC (WSOC) included. The muffin-tin radii of Au and Pt atoms were set equal to 1.402 and 1.333 Å, respectively. Inside each muffin-tin sphere, the basis functions were expanded in spherical harmonics with angular momentum up to $l_{max}=10$. The crystal wave functions were expanded into augmented plane waves with a cutoff of $k_{max}=4.0$ a.u., corresponding to the 139 basis LAPW functions per atom. For the presentation of the potential inside the muffin-tin spheres, $l_{max}^{pot}=8$ is used. Self-consistency was considered to be achieved when the electron density variation from iteration to iteration did not exceed $1\times 10^{-8}$ *e*/(a.u.)${}^{3}$. For self-consistent calculations, the irreducible part of the surface Brillouin zone (SBZ) was sampled using a (13×13×1) Monkhorst–Pack mesh [63] of k-points.

The lattice parameter of bulk platinum, calculated by minimizing the total energy, is 3.9788 Å, which is 1.4% larger than the experimental value (3.924 Å) [64]. The calculated lattice parameter of bulk gold is 4.1574 Å, which is 2.8% larger than the experimental value of 4.045 [65]. The lattice parameters obtained are consistent with the experimental ones [52,64,66,67,68,69,70,71,72] and agree well with other calculations [52,71,73,74,75] in which the PBE exchange-correlation potential was used.

The clean Pt(111) surface was modeled by a slab consisting of 41 Pt atomic layers. In the case of the *n*ML-Au/Pt(111) systems, the slab was covered on both sides with one, two, and three MLs of gold. To obtain the equilibrium geometry, the gold overlayers and four surface platinum layers on each side of the system were allowed to relax in the direction perpendicular to the slab plane. Atomic positions of 33 internal Pt atomic layers were frozen. The lateral lattice parameter of the Pt(111) surface (2.813 Å) was obtained from a bulk calculation with the same exchange-correlation functional, cutoffs, and a mesh with comparable k-point density. This value was kept for the lateral lattice constant in the *n*ML-Au/Pt(111) systems as well. The Au adsorbate lateral placement was chosen as a direct continuation of the fcc Pt lattice. This stacking better compares with the photoemission experiment [58]. The DFT calculations also show that this adsorption position is the most stable in the 1ML-Au/Pt(111) case [52].

## 3. Calculation Results and Discussion

### 3.1. Crystal Structure

**Figure 1:**
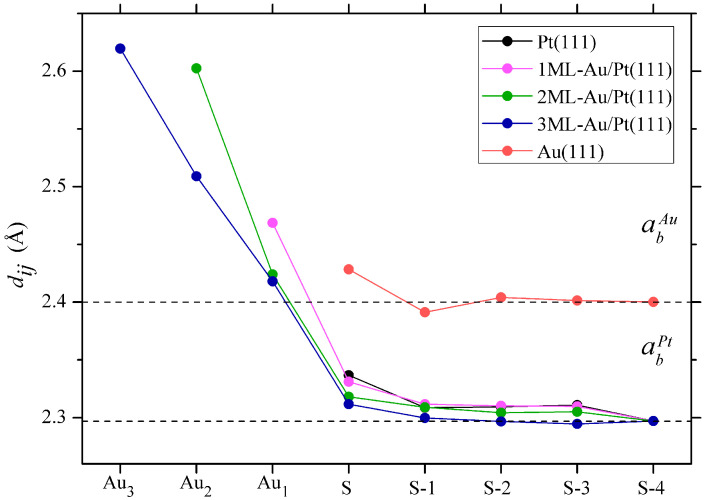
Relaxation of interlayer distances $d_{ij}$ of Au films and five surface layers of the platinum substrate (1ML—purple, 2ML—green, 3ML—blue), as well as five surface layers of pure gold (orange) and platinum (grey). The dotted lines show the values of interlayer distances in the direction (111) of bulk Pt and Au.

The calculated interlayer distances $d_{ij}$ and the relative relaxations $\delta_{ij}$ for all surfaces studied here are summarized in Table 1. The changes in the interlayer spacing in the substrate are presented with respect to the unrelaxed interlayer spacing $d_{o}^{Pt}$ = 2.297 Å of Pt, i.e., $\delta_{ij}=\left(d_{ij}-d_{o}^{Pt}\right)/d_{0}^{Pt}\times 100\%$. For $\delta_{ij}$ in the Au adlayer, we use the PBE calculated bulk parameter $d_{o}^{Au}$ = 2.400 Å. In the case of the Au-Pt interface spacing, we employ an average value $d_{o}=\left(d_{o}^{Pt}+d_{o}^{Au}\right)/2$. To make an easy analysis of the data in Table 1, they are presented graphically in Figure 1.

The crystal structure optimization of the Pt(111) slab shows that the relaxation of the first four surface layers is positive (upward relaxation). The obtained values are in good agreement with the results of LDA calculations [76]. A value of $\delta_{12}$ = 1.72% also agrees with that of 1.75% obtained in Ref. [77] for a 10 ML Pt(111) slab. The relaxation values of the first interlayer distance $\delta_{12}$ obtained in the low-energy electron diffraction (LEED) experiments (1.5 ± 0.9)% [78], (1.0 ± 0.1)% [67], and (1.1 ± 0.4)% [69] are in good agreement with our data. The spacings between deeper atomic layers are very close to the bulk ones [67,69,78]. Nevertheless, our spacing values are slightly higher in comparison with those of Ref. [77]. The latter can be explained by small values of $\delta_{ij}$ between those atomic layers. In general, the crystal structure of the clean Pt(111) surface determined here demonstrates good agreement with the experimental and theoretical data [66,67,68,69,70,71,72,73,77,79,80]. A detailed comparison of these data can be found, e.g., in Ref. [52].

When a gold ML is deposited on the Pt(111) surface, we find that the Au-Pt interlayer spacing is 2.469 Å, i.e., it is expanded by 5.12% concerning an ideal atomic geometry. Our value also exceeds the calculated one of 2.36 Å in Ref. [52]. Such a difference can be explained by a significantly larger slab employed in the present work. On the other hand, the interlayer spacing expansion between two top Pt atomic layers reduces to 1.47%. This value is very close to the expansion by 1.30% found in Ref. [52]. The distances between deeper Pt atomic layers are close to the bulk value within 1%. When the number of Au MLs increases to two, we observe that separation between two Au atomic layers increases to 2.602 Å. This corresponds to an expansion of 8.40% with respect to the Au bulk interlayer distance. By contrast, the Au-Pt interlayer spacing reduces to 2.424 Å. The interlayer expansion between the top two Pt MLs reduces by 0.91% as well. This is accompanied by a slight reduction in the $d_{ij}$ between the deeper Pt atomic layers. It can be explained by efficient screening by the Au MLs in these layers of the effects introduced by the presence of a surface. This tendency keeps holding with increasing the number of the Au MLs in the adlayer up to three. In 3ML-Au/Pt(111), the distance between top Au MLs increases to 2.62 Å, whereas *d* between the next two Au MLs reduces to 2.509 Å. The Au-Pt interlayer distance reduces to 2.418 Å as well. There is a reduction of the spacing between the first and second Pt atomic layers to 2.312 Å. The values of $d_{ij}$ for the deeper Pt atomic layers are now very close to their bulk value.

Please note that the crystal structure optimization of the Au(111) slab shows that the surface layer relaxation is 1.17% (upward relaxation). This is in good agreement with the value of 1.67% obtained in the calculations of Ref. [74]. As is known, a clean Au(111) surface does not have the (1 × 1) ideal periodicity and is reconstructed with a peculiar ∼(22×√3) incommensurate unit cell. However, employing the X-ray diffraction method, the authors of Ref. [81] experimentally found that the clean Au(111)-(1×1) surface experiences outward expansion by 1.5%. The DFT calculations performed in that work gave a value of 1.3% for the relaxation of the upper gold layer. Both values are in good agreement with our result for a clean Au(111) surface. However, these values are significantly lower in comparison to the interlayer distances between the surface Au atomic layer and the next atomic layer in the *n*ML-Au/Pt(111) systems found here. We explain such a difference by the usage of the Pt bulk lattice parameter in the plane parallel to the surface for all the systems studied. Since this parameter is about 4.5% lower in comparison to that for Au, the pronounced vertical expansion of the Au atomic layers seems reasonable. The buildup of strain in Au/Pt(111) associated with the Au/Pt lattice mismatch was deduced in [53].

### 3.2. Electronic Structure

#### 3.2.1. Clean Pt(111) and Au(111) Surfaces

**Figure 2:**
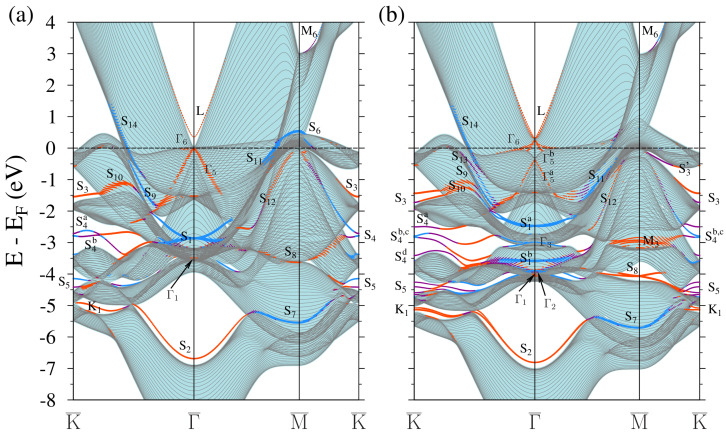
Electronic band structure of the clean Pt(111) surface calculated without (**a**) and with (**b**) inclusion of the spin-orbit interaction. The electronic states with strong localization in the top four atomic layers are highlighted by dots. The dot size is proportional to the level of this localization. The states with in-plane and out-of-plane polarization are marked by blue and red colors, respectively. Purple indicates surface states where there is no well-defined polarization. The Pt projected bulk band structure is represented by light-blue color regions.

**Figure 3:**
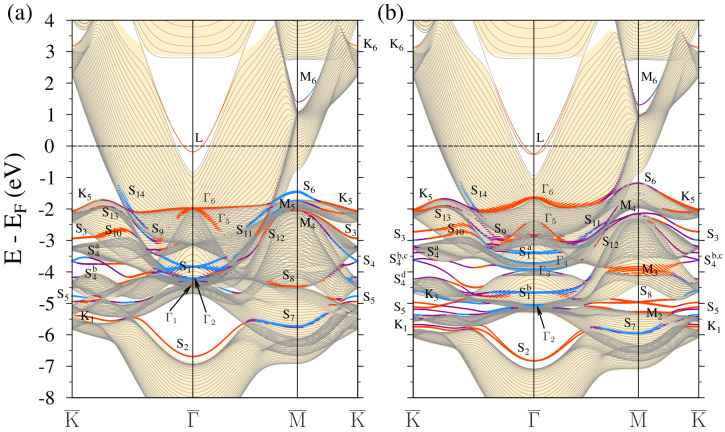
Electronic band structure of the clean Au(111) surface calculated without (**a**) and with (**b**) inclusion of the spin-orbit interaction. The electronic states with strong localization in the top four atomic layers are highlighted by dots. The dot size is proportional to the level of this localization. The states with in-plane and out-of-plane polarization are marked by blue and red colors, respectively. Purple indicates surface states where there is no well-defined polarization. The Au projected bulk band structure is represented by yellow color regions.

We start with a comparison of the calculated band structure of clean Pt(111) and Au(111) surfaces, which are presented in Figure 2 and Figure 3, respectively. The continuum of the Pt (Au) bulk states projected on this crystal face is highlighted by light-blue (yellow) regions. The slab bulk-like states representing this continuum are shown by thin grey solid lines. In our calculation employing a 41-ML thick slab, these lines are located significantly more densely in comparison to the previous calculations. Such dense representation helps us in resolving the dispersion of some surface states in more detail. It is especially helpful regarding the surface state labeled as L. For the surface states we have found, we adopt the notations used in [75,82]. We denote the surface states absent or not discussed in [75,82] by a symbol of the nearest symmetry point of the SBZ.

The presented figures demonstrate that the band spectra of the clean Pt(111) and Au(111) surfaces, calculated without (panels (a)) and with (panels (b)), taking into account the spin-orbit interaction (SOI), are similar to each other in principle. The main difference in these surfaces is the position of the Fermi level to the top of the *d* band. For instance, in the vicinity of $\bar{\Gamma }$, the *d* band top approaches the Fermi level in Pt(111), while in Au(111), it lies lower by more than 1.6 eV. The surface states and resonances of both surfaces are similar, with some exceptions. At $\bar{\Gamma }$, the gap bottom of the bulk states continuum (BSC) is completely above the Fermi level in Pt(111) and partially below the Fermi level in Au(111).

On Au(111), in this gap, there is a surface state labeled as L. It is well separated from the bulk states and formed mainly by the $p_{z}$ orbitals, with a small contribution of the *s* and $d_{3z^{2}-r^{2}}$ orbitals. On Pt(111), a similar surface state was found near the lower edge of the BSC gap. As seen in Figure 2b and Figure 3b, the switching on of the SOI leads to the Rashba splitting of this state. In this case, on the platinum surface, the lower edge of the BSC gap at $\bar{\Gamma }$ rises to 307 meV, and the intersection point of the Rashba surface states lies at 280 meV, i.e., 27 meV below the edge of the BSC gap. The energy of this state is in agreement with inverse photoemission [83] and time-resolved two-photon photoemission [84] experiments. Although at the $\bar{\Gamma }$ point, this state lies in the region of the BSC continuum, it has a pronounced surface character: 16% of its charge density located in a vacuum and 66% in the two upper layers. It is curious that on the gold surface, this state at the $\bar{\Gamma }$ point is localized somewhat weaker in the two upper layers (56%), despite that it is completely in the band gap. This L-gap surface state has been studied in detail in our previous work [59].

The second absolute gap at $\bar{\Gamma }$ on both surfaces contains the $S_{2}$ surface states. Extending practically along all symmetric directions of the SBZ, it merges the $S_{7}$ surface state in the vicinity of the $\bar{M}$ point and the $K_{1}$ surface state in the vicinity of the $\bar{K}$ point. At the $\bar{\Gamma }$ point, these states are localized in the two upper layers by more than 88% and formed by the *s* and $d_{3z^{2}-r^{2}}$ orbitals. In the WSOC case, at the $\bar{\Gamma }$ point at higher energy, there is also a strong S${}_{1}$ resonance in the symmetry gap. It is formed mainly by $d_{xy}$ and $d_{x^{2}-y^{2}}$ orbitals. The SOI splits these bands into a S${}_{1a}$–S${}_{1b}$ pair. Such states are present on both surfaces. They are localized in the surface layer by more than 88%. On the Pt(111) surface, the S${}_{1a}$ band, propagating along the $\bar{\Gamma }$-$\bar{K}$ direction, rises to the Fermi level and enters a small energy gap. At the boundary of this gap, it meets two sets of surface bands labeled as $S_{9}$ and $S_{10}$. The S${}_{1b}$ band propagates along the $\bar{\Gamma }$-$\bar{K}$ direction almost without dispersion. The S${}_{1a}$ and S${}_{1b}$ states have been observed and discussed previously [75,82,85]. Therefore, we do not discuss them in detail here.

In addition, we have found other surface states that have not been reported before. Near the SBZ center, these states are labeled as $\Gamma_{1}$–$\Gamma_{4}$. The $\Gamma_{5}$ and $\Gamma_{6}$ states found here were previously observed in an experiment and studied in [85]. The $\Gamma_{1}$ state is formed by $d_{3z^{2}-r^{2}}$ orbitals and is present only in the narrow vicinity of the $\bar{\Gamma }$ point. We have paid attention to this state because, in the case of the deposition of gold films on the surface of Pt(111), it falls into the energy gap of the substrate and forms a well-localized band of quantum well states (QWS) on the surface. The $\Gamma_{2}$ state appears to be a Tamm type state with the $d_{xy}$, $d_{x^{2}-y^{2}}$ symmetry. It splits off from a narrow region of a continuum of weakly dispersing bulk states of the $d_{xy}$, $d_{x^{2}-y^{2}}$ type (lying in the xy plane) into the region of the *s*, $p_{z}$, $d_{3z^{2}-r^{2}}$, and $d_{yz,zx}$ type bulk states (oriented along the *z* axis) with a rather large inverse dispersion. On the Au surface, 84% of its charge density is localized in the near-surface layer. On the Pt surface, 54% of this state is in the near-surface atomic layer and 22% in the fourth one from the surface.

The $\Gamma_{3}$ and $\Gamma_{4}$ surface resonances are observed only in the spectra obtained taking into account SOI. We found the $\Gamma_{4}$ resonance only on the gold surface. Both bands are formed by the $d_{xy}$, $d_{x^{2}-y^{2}}$ orbitals. Approximately 50% of its charge density is localized in the four surface layers. They are formed by the Tamm states, as they reside in the boundary of two bulk continuum regions of the different symmetries. The $\Gamma_{5}$ and $\Gamma_{6}$ surface resonances of a Tamm type are observed on both Pt and Au surfaces. These bands are formed by the $d_{yz,zx}$ type states detached from the bulk states continuum into a symmetry gap. On the Pt surface, the dispersion of these bands is almost linear. On the Au surface, it has quadratic dispersion of a hole type, i.e., their energy decreases with leaving the $\bar{\Gamma }$ point. Also note that on the platinum surface, these bands are weakly localized on the surface (not more than 45%), whereas on the gold surface, up to 90% of the charge density of this state is in the four surface layers.

In Figure 2b and Figure 3b, there are five (six) BSC gaps at the $\bar{K}$ point of the Pt (Au) surface band structure containing one or more surface states. These states are labeled by us as $S_{3}$, $S_{4}$ and $S_{5}$, as well as $K_{1}$, $K_{3}$, $K_{5}$, and $K_{6}$. The $S_{3}$ bands are two doubly spin degenerate ones formed by the $d_{yz,zx}$, $d_{xy}$, and $d_{x^{2}-y^{2}}$ orbitals in two surface layers with a clear predominance of the $d_{yz,zx}$ orbitals in the surface layer. The third (counting from the bottom) gap at the $\bar{K}$ point contains four $S_{4}$ surface bands. The $S_{4}^{a,b,c}$ bands are almost completely localized in the surface layer, while the $S_{4}^{d}$ band penetrates more deeply into the bulk region. Please note that the degree of localization of these states on the Pt surface is higher than on the Au one. The $S_{4}^{b,c}$ states are formed mainly by the $d_{yz,zx}$ orbitals with an admixture of the $d_{xy}$, $d_{x^{2}-y^{2}}$, and $p_{x,y}$ orbitals, while the $S_{4}^{a,d}$ states have the $d_{yz,zx}$, $d_{xy}$, and $d_{x^{2}-y^{2}}$ symmetry.

The second (from the bottom) BSC gap in the $\bar{K}$ point vicinity contains two doubly degenerate $S_{5}$ surface bands completely localized in four surface layers. Accounting for the SOC significantly increases the energy gap, especially on the gold surface, and splits both bands. The lower (in energy) of these two bands is localized mainly in the near-surface layer and is formed by $d_{xy}$, $d_{x^{2}-y^{2}}$ states with an admixture of the $d_{3z^{2}-r^{2}}$ type surface layer states. The upper band is localized mainly in the two surface layers and is formed by the $d_{yz,zx}$ type states with a significant admixture of the $d_{xy}$, $d_{x^{2}-y^{2}}$ states at the surface layer atoms. The latter band as a surface resonance has been reported in [75,82,85].

Also, we found several states which have not yet been discussed in the literature. On both surfaces, there is a $K_{1}$ surface state. On the Au(111) surface, two more states ($K_{3}$ and $K_{5}$) appear as well. The $K_{1}$ surface state is located at the center of the lowest band gap in the $\bar{K}$-$\bar{\Gamma }$ direction and is mainly formed by the $d_{yz,zx}$ orbitals of the second and third layer atoms from the surface, where almost 80% of the charge density of these states reside. These bands were observed in [75,82,85] but have not been discussed. Accounting for SOC leads to a splitting of this band into two degenerate spin bands. The lower band is localized in the near-surface layer, while the upper band is localized in the third layer from the surface. The $K_{3}$ surface state is observed only in the spectrum of the Au(111) surface calculated with taking into account the SOI. It has a resonance character and is formed by the $d_{xy}$, $d_{x^{2}-y^{2}}$ states with a significant admixture of the $d_{yz,zx}$ states of the surface layer atoms. The $K_{5}$ surface state extends along the bottom of the fifth band gap, and more than 75% of its charge is localized in two surface layers dominated by the $d_{3z^{2}-r^{2}}$ orbital with a small admixture of the $d_{yz,zx}$ contributions. The $K_{6}$ surface state is present in our figures on the Au(111) surface only. It is localized in two surface layers by 70%. Since most of this state is formed by a *s* orbital of the surface atom, it almost does not experience spin-orbit splitting.

At the $\bar{M}$ point, there are four BSC gaps on both surfaces, each containing several surface states. Without accounting for SOI, the $S_{6}$ surface state is localized in the surface layer by more than 90%. Accounting for SOI significantly reduces its surface character on the Au(111) surface and destroys it on the Pt(111) surface. The $S_{7}$ surface state has the $d_{x2-y2}$ type symmetry with an admixture of the *s* states. Despite its resonance character, the charge density of this state is more than 72% localized in the surface layer. Without accounting for SOI, the $S_{8}$ surface state lies in the bulk continuum and has a resonant character. Taking SOI into account opens a band gap in the projection of the bulk spectrum, into which this state falls. Also, it experiences a spin-orbit splitting of a Rashba type. As a result, it is localized in the two surface layers by more than 88%.

In addition, we found at the $\bar{M}$ point several other surface states not discussed earlier. These are the $M_{2}$, $M_{3}$, $M_{4}$, $M_{5}$, and $M_{6}$ states. The $M_{2}$ state is present only on the Au(111) surface in the spectrum calculated considering SOI. It is mainly localized in the third and fourth layers from the surface (75%) and is formed by the $d_{yz,zx}$, $d_{3z^{2}-r^{2}}$ type orbitals with a noticeable admixture of the $d_{x^{2}-y^{2}}$ orbitals. The $M_{3}$ surface state is observed on both surfaces when SOI is taken into account only. It is formed by the $d_{yz,zx}$ type states and has a pronounced resonance character with localization near the surface atomic layer up to 66%. The surface states $M_{4}$ and $M_{5}$ are observed on the Au(111) surface. They are formed by the $d_{xy}$ and $d_{3z^{2}-r^{2}}$ type orbitals and have a resonant character (50% in the four surface layers). The inclusion of the SOI destroys the former state, as can be deduced from the comparison of Figure 3a,b. The $M_{6}$ surface band is strongly localized in the two surface layers (96%). It has a complex orbital composition dominated by the $p_{x}$ symmetry with notable contributions from the *s*, $d_{x^{2}-y^{2}}$, and $d_{3z^{2}-r^{2}}$ orbitals. To conclude this paragraph, we note that most of the surface states found here are well recognized in the photoemission spectra presented in [85].

#### 3.2.2. *n*ML-Au/Pt(111) Systems

Gold adsorption on the Pt(111) surface significantly alters its surface electronic properties. In general, the main changes consist of the appearance of Au quantum well states with strong localization in the adlayer, the emergence of Au surface states, and the Au/Pt interface states. Based on this, we will analyze the states with strong localization in the adlayer and the nearby substrate region. The surface states associated with the uppermost atomic layers of Au and Pt are indicated by the respective superscripts. The Au quantum well states are denoted by the symbols $\bar{\Gamma }$, $\bar{K}$, and $\bar{M}$. The states denoted by the symbol *I* correspond to the Au/Pt interface states.

**Figure 4:**
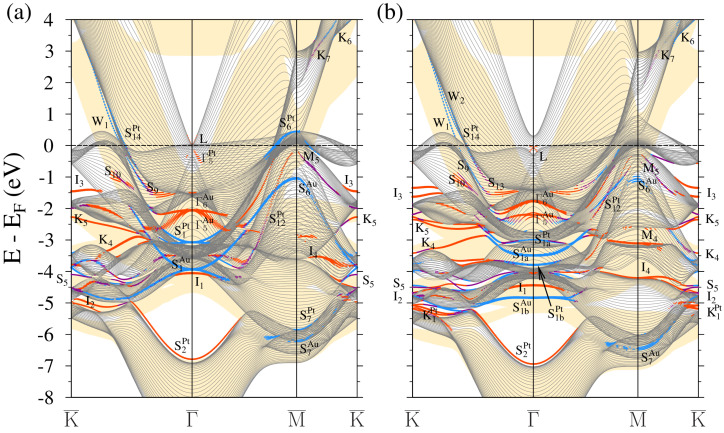
Electronic band structure of the 1ML-Au/Pt(111) surface calculated without (**a**) and with (**b**) inclusion of the spin-orbit interaction. The electronic states with strong localization in the top four atomic layers are highlighted by dots. The dot size is proportional to the level of this localization. The states with in-plane and out-of-plane polarization are marked by blue and red colors, respectively. Purple indicates surface states where there is no well-defined polarization. The Au projected bulk band structure is represented by yellow color regions.

In Figure 4, where the electronic structure of 1ML-Au/Pt(111) is presented, we observe many electronic states with localization in the surface region. The most prominent consequence of a 1ML-Au adsorption is a strong modification of the *L* surface state. Its evolution from a true surface state of a clean Pt(111) to a resonance surface state of a 1ML-Au/Pt(111) heterostructure was traced in Ref. [59]. At the finite wave vectors (∼0.1 Å${}^{-1}$), it has a linear dispersion. In the WSOC case of Figure 4a, the *L* surface state lies above the Fermi level on the lower boundary of the bulk band gap. Accounting for the spin–orbital interaction raises the lower edge of the bulk band gap in the vicinity of the $\bar{\Gamma }$ point. As a result, the *L* state lies in the background of the Pt bulk band continuum. The latter circumstance may make it difficult for experimental detection of this state. A linear Dirac-cone-like energy dispersion of the *L* surface state produces a situation when only the upper cone reaches the Fermi level. In consequence, this surface can be interesting for conversion between spin and charge currents through the Edelstein effect [86,87,88].

**Figure 5:**
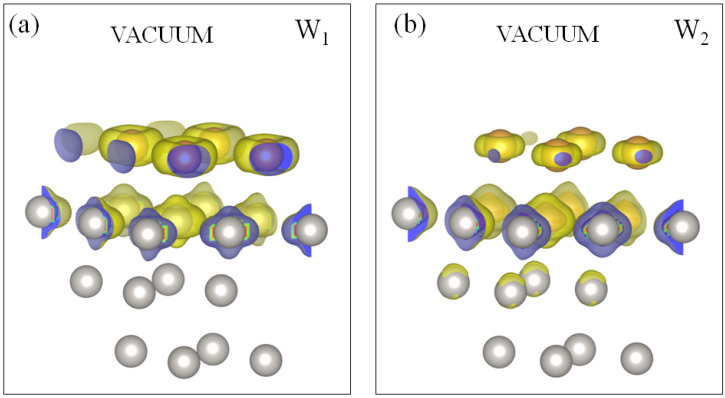
Charge density distribution for the Au quantum well states (**a**) $W_{1}$ and (**b**) $W_{2}$ at the energy of 0.5 eV above the Fermi level in the 1ML-Au/Pt(111) system. Positions of the Au (Pt) atoms are represented by yellow (grey) circles.

In the energy region above the Fermi level, in Figure 4a we find the gold-induced QWS $W_{1}$ of a *s*-*p* character in the Pt energy gap strongly dispersing along the $\bar{\Gamma }$-$\bar{K}$ direction on the background of the bulk states projection of gold. The latter circumstance indicates that following an increase in the coating thickness, such states represent quantization in the direction perpendicular to the surface plane of a continuum of the projected bulk states of gold. As seen in Figure 4b, the switching on SOI splits this QWS into two branches $W_{1}$ and $W_{2}$. In Figure 5, we report the spatial distribution of the charge density of these states. One can see that they are strongly confined to the two surface atomic layers. In the case of $W_{1}$, the charge is almost evenly distributed between the gold and the top platinum atomic layers. As for the $W_{2}$ state, its charge maximum concentrates in the top Pt atomic layer. This can be explained by the proximity of this state to the Pt bulk continuum. In the vicinity of the Fermi level, these QWSs experience strong hybridization with the Pt *d* bulk-like states and can hardly be followed. Along other directions, there are no such states linked to the gold adlayer. Only weak resonances can be detected at energies above ∼1.5 eV along the $\bar{M}$-$\bar{K}$ direction.

Due to the strong confinement of the $S_{1a}$ and $S_{1b}$ surface states to the Pt and Au atomic planes, in 1ML-Au/Pt(111), we observe two sets of such states shown in Figure 4a. The bands labeled as $S_{1a}^{Pt}$ and $S_{1b}^{Pt}$ are mainly confined to the top Pt atomic layers. The similar bands with strong localization in the gold ML are marked as $S_{1a}^{Au}$ and $S_{1b}^{Au}$. Due to a shallow dispersion, these states contribute substantially to the local density of states in the corresponding atomic layers.

The deposition of a gold ML results in a slight downward shift of the $S_{2}$ surface state. In Figure 4b its bottom locates at −6.9 eV at $\bar{\Gamma }$. Spatially, its wave function has large localization in the top three atomic layers with a strong presence in the Pt substrate.

The dispersion of the platinum $S_{3}$ surface states is not affected by the gold ML adsorption at all, as is seen in Figure 4a,b. This can be explained by its energy position in the energy gaps of both the Pt(111) and Au(111) surfaces. On the other hand, its charge density in the 1ML-Au/Pt(111) system penetrates substantially into the gold adlayer. For this reason, we classify these states in the vicinity of the $\bar{K}$ point as an interface state $I_{3}$.

At $\bar{K}$, we find the QWSs $K_{1}$, $K_{4}$, and $K_{5}$ at lower energies with strong localization in the Au atomic layer. An interface state $I_{2}$ is evenly distributed in the gold and the top platinum atomic layers.

**Figure 6:**
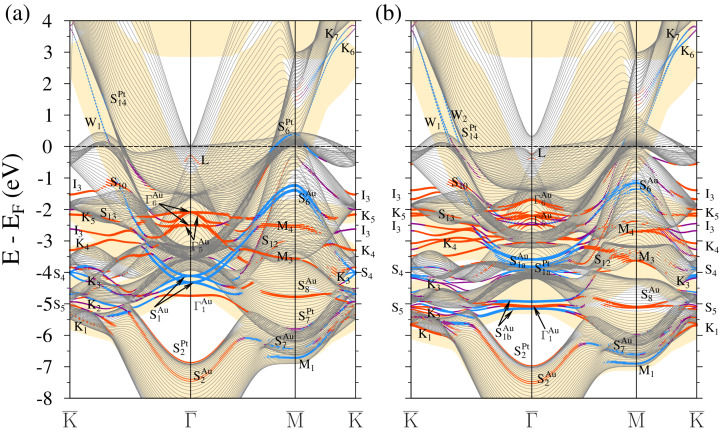
Electronic band structure of the 2ML-Au/Pt(111) surface calculated without (**a**) and with (**b**) inclusion of the spin-orbit interaction. The electronic states with strong localization in the top four atomic layers are highlighted by dots. The dot size is proportional to the level of this localization. The states with in-plane and out-of-plane polarization are marked by blue and red colors, respectively. Purple indicates surface states where there is no well-defined polarization. The Au projected bulk band structure is represented by yellow color regions.

Increasing the number of Au MLs deposited on Pt(111) up to two MLs results in the SOC electronic structure shown in Figure 6b. The corresponding WSOC case is reported in Figure 6a. The number of gold-induced states increases considerably. As for the *L* surface state, due to strong interaction with the Pt substrate states, in Figure 6b, it presents a typical resonance-like behavior with a center of gravity located at −0.25 eV at $\bar{\Gamma }$. Like in the previous case, the upper branch has a positive dispersion and almost reaches the Fermi level.

**Figure 7:**
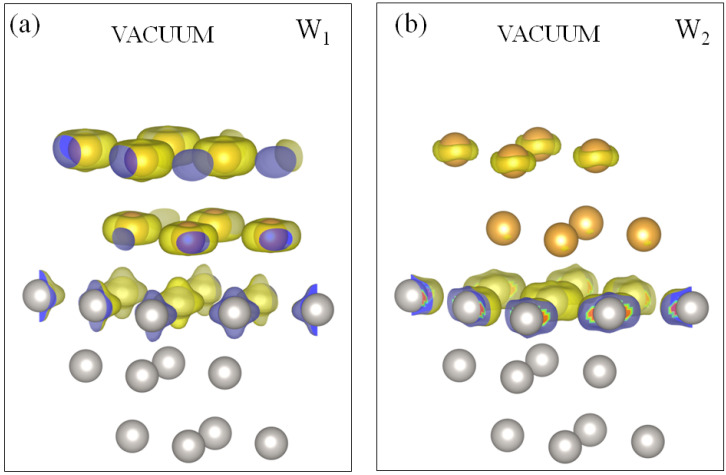
Charge density distribution for the Au quantum well states (**a**) $W_{1}$ and (**b**) $W_{2}$ at the energy of 0.5 eV above the Fermi level in the 2ML-Au/Pt(111) system. Positions of the Au (Pt) atoms are represented by yellow (grey) circles.

Above the Fermi level, in Figure 6b, we observe in the Pt energy gap along the $\bar{\Gamma }$-$\bar{K}$ direction the gold QWSs labeled as $W_{1}$ and $W_{2}$ of mainly *s*-*p* character. In Figure 7, we present the charge density distribution of these states. It is seen that they are almost entirely confined to the top three atomic layers. The $W_{1}$ state density has maximum localization in the two gold atomic layers, whereas the $W_{2}$ state is strongly confined to the top platinum layer. In this system, both these states disperse up to the $\bar{K}$ point, transforming into the true surface states. Upon departure from $\bar{K}$ toward the $\bar{M}$ point, these bands disperse downward but disappear without reaching the Fermi level. Along $\bar{\Gamma }$-$\bar{M}$, this system does not support such states. Only in the vicinity of the $\bar{M}$ point a hole-like gold band with the top at 0.3 eV can be detected.

Regarding the states similar to the $S_{1a}$ and $S_{1b}$ surface states, in 2ML-Au/Pt(111), we find again two sets of such states. The dispersion of the Pt-derived $S_{1a}^{Pt}$ and $S_{1b}^{Pt}$ states shows some variation in comparison to the 1 Au ML case of Figure 4b. Even more variation is observed in the dispersion of the gold-induced $S_{1a}^{Au}$ and $S_{1b}^{Au}$ states. This seems reasonable since the gold-induced electronic states should be more sensitive to the number of MLs in the adlayer.

A somehow different picture we observe in the low-energy part of Figure 6a,b. Here, we detect two $S_{2}$-like energy bands. The wave function of the upper energy band $S_{2}^{Pt}$ is linked to the Pt substrate, whereas the lower $S_{2}^{Au}$ band presents strong localization in the Au adlayer. Like in the 1ML-Au(111) system, the $S_{3}^{Pt}$ states are not affected by the adlayer in 2ML-Au/Pt(111), as is evident from its dispersion in Figure 6a,b.

With two gold atomic layers deposited, the classification of the other states with strong localization in the surface region becomes more certain. Thus, in Figure 6 at the $\bar{K}$ point we observe the QWSs $K_{1}$, …, $K_{7}$ being the representatives of the respective Au bulk bands. Nevertheless, some of them present a resonance character since they reside in the energy regions where the Pt bulk states exist. Additionally, the states resembling the states $S_{4}$ and $S_{5}$ of the Au(111) surface can be detected as well.

As for the interface state $I_{3}$ found in the 1ML-Au/Pt(111) system, in Figure 6a, such a state with a similar dispersion is observed as well. Other interface states labeled as $S_{3}$ are found at lower energies. We trace their origin to the state $S_{3}$ on the clean gold surface.

**Figure 8:**
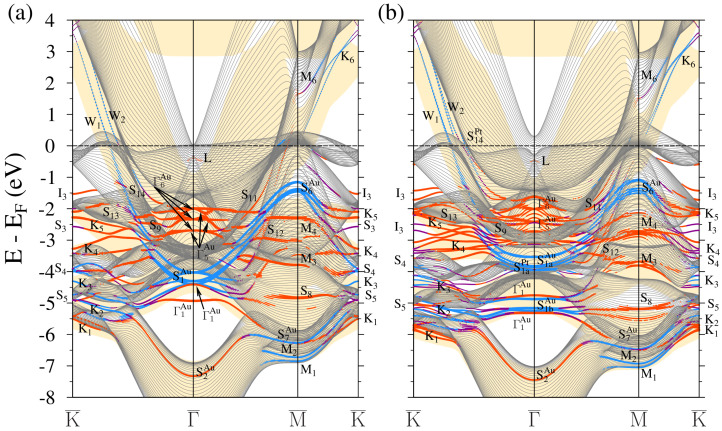
Electronic band structure of the 3ML-Au/Pt(111) surface calculated without (**a**) and with (**b**) inclusion of the spin-orbit interaction. The electronic states with strong localization in the top four atomic layers are highlighted by dots. The dot size is proportional to the level of this localization. The states with in-plane and out-of-plane polarization are marked by blue and red colors, respectively. Purple indicates surface states where there is no well-defined polarization. The Au projected bulk band structure is represented by yellow color regions.

The electronic structure of 3ML-Au/Pt(111) is reported in Figure 8. Like in the previous systems, the WSOC and SOC cases are presented in panels (a) and (b), respectively. The *L* surface state with strong resonance character [59] has the energy of −0.4 eV at the $\bar{\Gamma }$ point, i.e., very close to its value on a clean Au(111) surface. Nevertheless, its dispersion is strongly affected by the Pt bulk-like states. Upon departure from the $\bar{\Gamma }$ point, its dispersion can be traced in a small region of the SBZ only.

**Figure 9:**
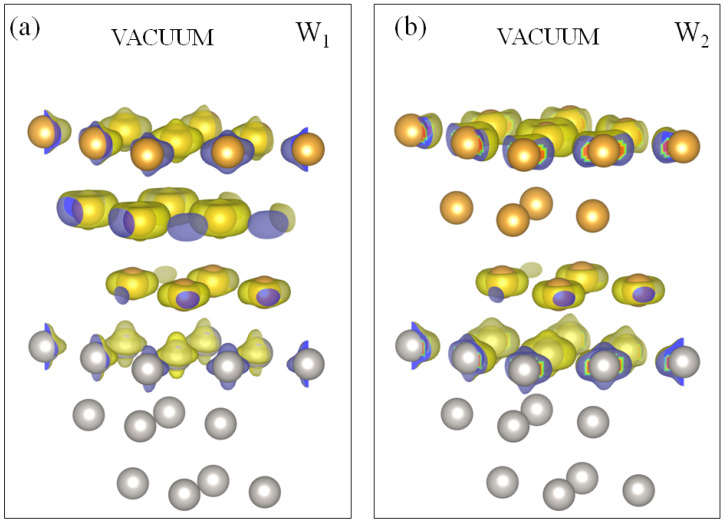
Charge density distribution for the Au quantum well states (**a**) $W_{1}$ and (**b**) $W_{2}$ at the energy of 0.5 eV above the Fermi level in the 3ML-Au/Pt(111) system. Positions of the Au (Pt) atoms are represented by yellow (grey) circles.

In the SOC spectrum of Figure 6b, above the Fermi level along the $\bar{\Gamma }$-$\bar{K}$ direction, we observe two gold-derived QWSs of *s*-*p* character $W_{1}$ and $W_{2}$. Curiously, such states appear again in the Pt energy gap only. A large portion of the wave-vector–energy phase space where the bulk-like states in Au(111) can exist (yellow region) is free of such states. As seen in Figure 9a, the charge density of the $W_{1}$ QWS strongly localizes in the Au adlayer with a maximum in the middle atomic layer. In the top Pt atomic layer, its small portion resides only. In the case of the $W_{2}$ state, the charge density shown in Figure 9b localizes in the first and third gold atomic layers. A notable part resides in the top Pt layer as well. Like in 2ML-Au/Pt(111), both states reach the $\bar{K}$ point transforming into true surface states. In the $\bar{K}$-$\bar{M}$ direction, these bands disperse downward without reaching the Fermi level. Along $\bar{\Gamma }$-$\bar{M}$, this system does not support similar states.

Notice that in all three Au/Pt(111) systems studied, we found two gold QWSs in the vicinity of the Fermi level presenting strong localization in the gold adlayer and the platinum top atomic layer. Strong localization of quantum states in thin adsorbate layers might be interesting in the field of plasmonics [89,90]. The presence of such states may lead to the realization of low-energy collective electronic excitations (plasmons) [91,92] characterized by slow propagation velocities and much stronger spatial confinement in comparison to conventional surface plasmon polaritons [93,94,95]. In these systems, such plasmons might be much stronger in comparison to conventional acoustic surface plasmons based on the metal surface states in noble metals [96,97,98] and comparable to those found in the strongly doped graphene systems [99,100,101].

The classification of the surface-derived states in the band structure of 3ML-Au/Pt(111) below the Fermi level is essentially similar to that in the case of the 2ML-Au/Pt(111) system. The QWSs become defined more clearly, as well as the gold surface states. The only exception is the lower $I_{3}$ state. In the 3ML-Au/Pt(111) system, it is still an interface state that has dispersion different from that of the $S_{3}$ surface state on the clean Au(111) surface. In Figure 8b, we can still detect the $S_{1a}^{Pt}$ and $S_{1b}^{Pt}$ states with significant localization in the top Pt atomic layer. The corresponding gold surface states $S_{1a}$ and $S_{1b}$ have dispersion close to that in the 2ML-Au/Pt(111) case, signaling a complete formation of these Au surface states in such a thin adsorbed film.

### 3.3. Work Function

The work function of the metal is defined as a difference between the vacuum level position and the Fermi level. Since both these quantities are obtained in the DFT calculations, the work function values can be directly evaluated in our case.

Initially, analysis of tendencies of the work function variation in thin metal films was based on a free-electron-like jellium model [102]. Later, a quantitative analysis based on first-principles density-functional calculations became available [41]. A basic picture was established by studying a jellium model [102]. The position of the Fermi level with respect to the vacuum level varies with changes in the film thickness, which is ultimately determined by the number of occupied sub-bands, frequently called QWSs, in the film. The number of such sub-bands increases as the thickness of the film increases. Whenever a new sub-band crosses the Fermi level, the calculated work function exhibits a cusp.

However, the case studied here is different in at least two important aspects. First, the projected bulk band structure of Au(111) has a wide energy gap at the Fermi level at the SBZ center. As a result, QWSs in thin Au(111) films experience severe constraints in their energy positions. Second, at the Fermi level, the electronic structure of a Pt(111) substrate has an energy gap only in a small region around the $\bar{K}$ point. In consequence, strong hybridization of the gold-induced QWS bands with the Pt bulk-like states is possible.

**Figure 10:**
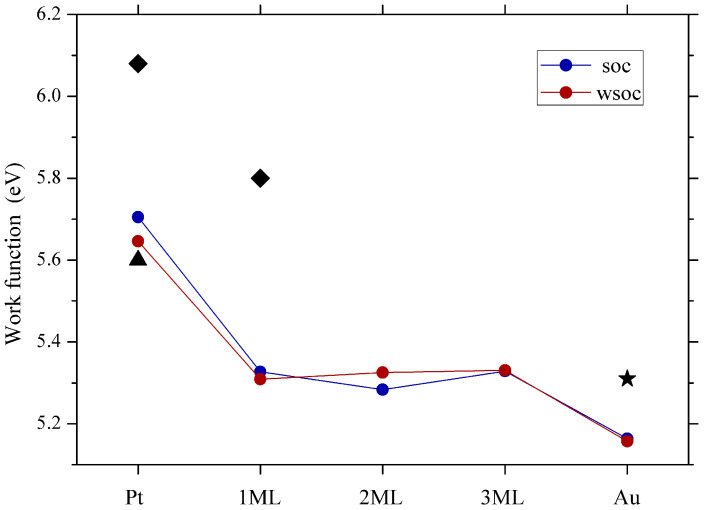
Work function of the (111) surface of Pt, Au, and the *n*ML-Au/Pt(111) system calculated without and with taking into account the SOI. The experimental data for Pt(111) and 1ML-Au/Pt(111) [49], Pt(111) [103], and Au(111) [104] are shown by diamonds, triangle, and star, respectively.

Our calculations show that the work function of a clean Pt(111) surface drops significantly upon the adsorption of one gold ML. It changes by 0.34 eV from 5.65 eV in Pt(111) to 5.31 eV in 1ML-Au/Pt(111). The work function variation of Pt(111) as a function of Au coverage was studied in Ref. [49]. It was found that the work function decreased upon gold deposition from its initial value by 0.28 eV at a 1 ML coverage, which correlates with our findings and remained constant thereafter. However, as is seen in Figure 10, in the system with two gold MLs, the work function reduces to 5.32 eV. The subsequent increase in the Au adlayer thickness to three MLs results in an increase of the work function to 5.33 eV. In the experimental data of Ref. [49], such behavior was not established, probably due to variation of the measured data in about 0.1 eV in this coverage region. For comparison, in Figure 10, we report the data obtained in the calculations without taking into account the SOI. It is seen that its inclusion varies the calculated values on a 0.05 eV scale.

Such behavior of the work function value with variation in the film thickness was observed in several systems. Oscillations in the WF on a few tens meV energy scale in *n*ML-Au/Pt(111) is significantly smaller in comparison to a hundred meV variation in the case of thin Pb films [41]. This is probably a reason why, in *n*ML-Au/Pt(111), such variations were not resolved in the experiment [49]. In the *s*-*p* systems like Pb thin films, the work function changes were explained by the variation of the energy position of the energy bands crossing the Fermi level with changing the film thickness. In the *n*ML-Au/Pt(111) system, such analysis is difficult to perform since clear *s*-*p* gold-induced QWSs in the vicinity of the Fermi level are observed in the energy gap along the $\bar{\Gamma }$-$\bar{K}$ direction only.

Please note that our calculated work function of the pure Pt(111) surface is close to the lower limit of the range of work function values obtained experimentally (5.6 and 6.1 eV [49,103,105,106,107,108,109]). Our calculated value of the pure Au(111) surface work function of 5.16 eV is lower in comparison to the experimental results (5.31 to 5.55 eV [104,110,111]). However, we note that our value is in good agreement with other ab initio calculations [112,113].

## 4. Conclusions

We studied the atomic crystal and electronic structures of the thin gold films adsorbed on the Pt(111) surface. For comparison, similar calculations were realized for the clean Au(111) and Pt(111) surfaces. The spin-orbit interaction was taken into account. The calculated electronic structure of the *n*ML-Au/Pt(111) systems with *n* = 1, 2, 3 allowed us to trace the appearance and evolution of the gold-induced quantum well states with the variation of *n*. We also follow the appearance of the gold-induced surface states. The formation of the majority of all these states can be recognized in the systems with n≥2. Such thin thickness of the gold adlayer required for the appearance of the quantum well states can be explained by their predominantly *d* character. We found that the *s*-*p* surface state *L* state becomes occupied at the surface Brillouin zone center upon deposition of Au adlayer of any thickness. The predicted effects can be observed by the angle-resolved photoemission, inverse two-photon photoemission, and time-resolved photoemission.

The calculated work function presents oscillating behavior with variation in the number of atomic layers in the Au adlayer. The inclusion of the spin-orbit interaction has some impact on the calculated values of the work function. Nevertheless, the effect is limited to a few tens meV, i.e., notably smaller in comparison to the free-electron metal case.

## Figures and Tables

**Table 1:** Relaxed interlayer distances $d_{ij}$, relative relaxations $\delta_{ij}$ for the clean Pt(111) surface and *n*ML-Au/Pt(111) systems. The subscripts *i* and *j* number the atomic layers, starting from the Au-Pt or vacuum-Pt interfaces. *L* denotes the sort of the atomic layer.

$L_{i}$-$L_{j}$	Pt(111)		1ML-Au/Pt(111)		2ML-Au/Pt(111)		3ML-Au/Pt(111)	
	$d_{\mathrm{ij}}$ **(Å)**	$\delta_{\mathrm{ij}}$ **(%)**	$d_{\mathrm{ij}}$ **(Å)**	$\delta_{\mathrm{ij}}$ **(%)**	$d_{\mathrm{ij}}$ **(Å)**	$\delta_{\mathrm{ij}}$ **(%)**	$d_{\mathrm{ij}}$ **(Å)**	$\delta_{\mathrm{ij}}$ **(%)**
Au${}_{3}$-Au${}_{2}$	−	−	−	−	−	−	2.620	9.15
Au${}_{2}$-Au${}_{1}$	−	−	−	−	2.602	8.40	2.509	4.53
Au${}_{1}$-Pt${}_{1}$	−	−	2.469	5.12	2.424	3.21	2.418	2.95
Pt${}_{1}$-Pt${}_{2}$	2.337	1.72	2.331	1.47	2.318	0.91	2.312	0.63
Pt${}_{2}$-Pt${}_{3}$	2.309	0.50	2.312	0.63	2.309	0.52	2.300	0.11
Pt${}_{3}$-Pt${}_{4}$	2.309	0.53	2.310	0.57	2.304	0.31	2.297	−0.02
Pt${}_{4}$-Pt${}_{5}$	2.311	0.60	2.310	0.55	2.305	0.35	2.295	−0.11

## Data Availability

Data are contained within the article.
